# Establishment of the Volatile Signature of Wine-Based Aromatic Vinegars Subjected to Maceration

**DOI:** 10.3390/molecules23020499

**Published:** 2018-02-23

**Authors:** Rosa Perestrelo, Catarina L. Silva, Pedro Silva, José S. Câmara

**Affiliations:** 1 CQM—Centro de Química da Madeira, Universidade da Madeira, Campus da Penteada, 9020-105 Funchal, Portugal; rmp@uma.pt (R.P.); cgsluis@uma.pt (C.L.S.); pedro_dasilva@hotmail.com (P.S.); 2 Departamento de Química, Faculdade de Ciências Exatas e Engenharia, Universidade da Madeira, Campus da Penteada, 9020-105 Funchal, Portugal

**Keywords:** vinegars, volatile signature, HS-SPME/GC-MS, PCA, authenticity

## Abstract

The flavoring of vinegars with aromatic fruits and medicinal herbs is a practice with increasing trend mostly in countries with oenological tradition, resulting in a product of improved quality and consumer attractiveness. This study was directed towards the evaluation of the impact of the maceration process on the volatile signature of wine-based aromatic vinegars (WBAVs). The evaluation was performed using solid phase microextraction (SPME) combined with gas chromatography combined with mass spectrometry (GC-MS). Experimental parameters influencing headspace solid (HS)-SPME extraction efficiency, were optimized using an univariate experimental design. The best results were achieved using a polydimethylsiloxane (PDMS) fiber, 10 mL of vinegar sample, at 50 °C for 30 min of extraction. This way One hundred and three volatile organic compounds (VOCs), belonging to different chemical families including ethyl esters (37), higher alcohols (20), fatty acids (10), terpenoids (23), carbonyl compounds (six), lactones (five) and volatile phenols (two), were identified in wine vinegar (control) and WBAV. As far as we know, 34 of these VOCs are reported for the first time in macerated vinegars. Higher alcohols and lactones are the major chemical families in WBAV macerated with apple, whereas terpenoids are predominant in WBAV macerated with banana. The obtained data represent a suitable tool to guarantee the authenticity and genuineness of WBAV, as well as to promote the production of WBAV with improved sensorial and organoleptic properties. To the best of our knowledge, there are no reported studies dealing with the volatile signature of WBAV enriched with banana, passion fruit, apple and pennyroyal.

## 1. Introduction

**Figure 1:**
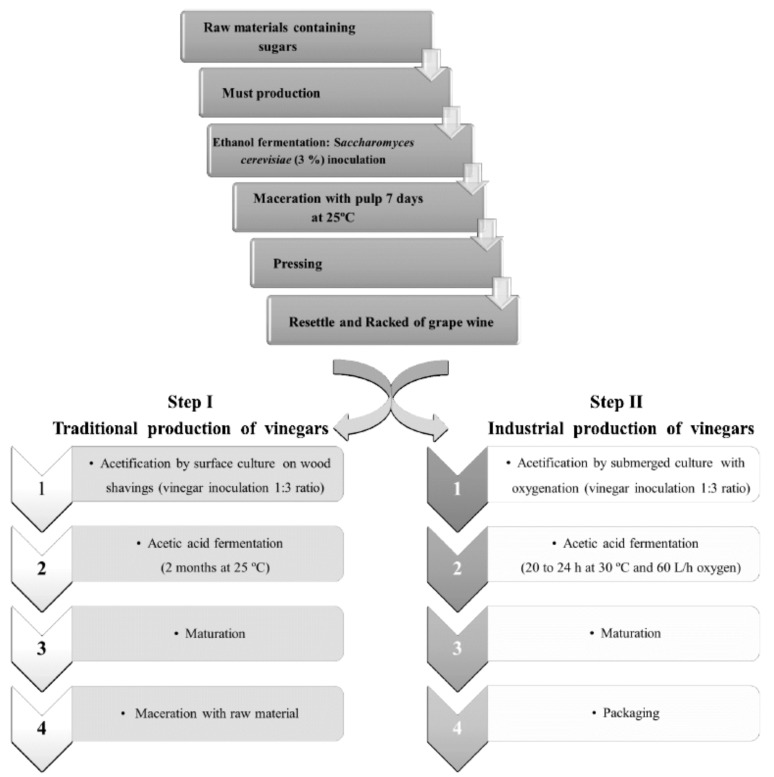
Flowchart showing the general production methods for vinegar [2,3,4].

Vinegar is a traditional food product with a high reputation throughout the world, used not only as a condiment, but also as preserving agent for a wide range of foods [1]. It is produced from raw materials containing mainly carbohydrates in two-stage fermentation processes, where the first one involves ethanol formation by yeasts (usually *Saccharomyces* species) through the conversion of fermentable sugars (alcoholic fermentation) and subsequently the oxidation of ethanol to acetic acid (acetification). From a technological point of view, there are mainly two defined vinegar making methods (Figure 1): (*i*) the traditional method, in which the culture of acetic acid bacteria is placed on the surface of a barrel in a direct contact with oxygen; and (*ii*) the industrial method, a quick process involving a submerged culture where the oxygenation has been greatly improved [2,3,4].

The market is turning towards the diversity of food and ingredients that prevent several diseases, such as those showing antibacterial, anti-inflammatory, blood glucose control, lipid metabolism regulation, weight loss, anticancer and cardiovascular disease prevention activity [2,5,6,7]. Taking into account these reasons, the agri-food market is focused on the development of novel products based on traditional processes with higher nutritional and organoleptic properties. Following the research line of development of products with increased value from a nutritional point of view, a new sherry vinegar-derived product enriched with dietary fiber has been developed by Marrufo-Curtido et al. [8]. Venturi et al. [9] developed a phenol-enriched refined olive oil with its own phenolic compounds extracted from wastewater produced during physical processing in order to improve the nutraceutical value, as well as the antioxidant capacity of olive oil. On the other hand, marine sources (e.g., macroalgae, microalgae), food by-products and plant-derived natural products can be used to extract bioactive compounds, such as polyunsaturated fatty acids (PUFAs) and phenolics, among others, that are widely recognized as important nutritional components that may help prevent various cardiac disorders [10,11,12,13].

Recently, new products associated with several fruits have arisen, such as vinegars macerated with fruits, fruit juice with added vinegar and fruit vinegars, which improve the organoleptic and health-promoting characteristics [5,6,7,14,15,16,17]. Its quality and acceptance by consumers depends on several parameters, being aroma one of the most important. In wine-based vinegars and derived products, aroma results from several hundreds of volatile organic compounds (VOCs) belonging to different chemical families (e.g., mono- and sesquiterpenes, esters, higher alcohols, carbonyl and sulphur compounds) encompassing a wide range of volatilities and polarities. These VOCs may come from the raw materials (e.g., red wines, fruits, cider, malted barley, honey, among others), and/or may be formed during production and storage processes [1,4,6,14,15,16,17,18]. VOCs are present, to a large extent, in fruits and aromatic/medicinal herbs influencing positively the final quality of vinegars by the addition of new compounds derived from them. In addition, fruits are rich in vitamins, minerals, phytochemicals, and contain potentially bioactive compounds including polyphenols (e.g., flavonoids and non-flavonoids) which have been shown to prevent oxidative processes. These compounds confer to fruits a significant antioxidant capacity related to numerous healthy properties [2,5,6,7].

Due to the diversity of commercially available vinegars and vinegar-based products and its increasing demand trend, the development of reliable and high throughput analytical methodologies able to establish criteria for determining its quality and origin are crucial to achieve this purpose [19]. Several extraction techniques, such as steam distillation [20], liquid-liquid extraction [21], stir bar sorptive extraction [5], solid-phase extraction [22], and solid-phase microextraction (SPME) [23] have been applied to isolate the volatiles from vinegars and vinegar-derived products. Compared to other extraction processes SPME offers several advantages such as the fact it eliminates the use of extraction solvent and allows the extraction and the concentration steps to be performed simultaneously [23]. The vinegar volatile signature is usually established using chromatographic techniques, in particular gas chromatography combined with mass spectrometry (GC-MS) [24].

The characterization and discrimination of vinegars have been widely investigated through the integration of chromatographic data with chemometric tools, such as principal component analysis (PCA) and hierarchical cluster analysis (HCA). PCA and HCA are among the most used chemometric tools to view, analyze and explore the large information provided by the analytical instrumentation. These two pattern recognition tools complement one another and have been widely applied to solve classification problems [25,26].

Following the research line of novel aromatic vinegars, with improved nutritional and sensorial properties, the aim of the current work was the establishment of he volatile signature of a wine vinegar (control) and wine-based aromatic vinegars (WBAVs) macerated with different fruits (e.g., banana, passion fruit and apple) and a medicinal herb (e.g., pennyroyal) using headspace solid-phase microextraction (HS-SPME) combined with gas chromatography-mass spectrometry (GC–MS). In addition, chemometric tools, namely PCA and HCA, were applied to obtain deep insights into variations on the volatile signature of target vinegars, and to identify the VOCs responsible for the discrimination among vinegars. Our study’s findings could provide to “home-made” producers new opportunities to promote the production of wine vinegars with enhanced levels of odoriferous VOCs, to guarantee the vinegars typicality and to expand the oenological market. To the best of authors’ knowledge, there has not been any report on the volatile signatures of WBAV macerated with banana, passion fruit, apple and pennyroyal.

## 2. Results and Discussion

### 2.1. HS-SPME Optimization

To develop a suitable and powerful HS-SPME/GC-MS method in order to establish the volatile signature of wine vinegar (control) and WBAV (banana, passion fruit, apple and pennyroyal), the most important experimental parameters influencing the HS-SPME extraction efficiency, including fiber coating, extraction time, extraction temperature, sample volume, ionic strength and desorption time, were optimized using an univariate experimental design. The optimal extraction conditions were chosen based on total GC peak area, number of isolated and identified VOCs and precision (expressed as relative standard deviation, % RSD).

#### 2.1.1. Fiber Coating

The selection of the most suitable SPME fiber depends on the composition of the sample material under study [27]. Five fiber coatings, including carboxen/polydimethylsiloxane (CAR/PDMS, 75 μm), divinylbenzene/carboxen on polydimethylsiloxane (DBV/CAR/PDMS; StableFlex, 50/30 μm), polyacrylate (PA, 85 μm), polydimethylsiloxane (PDMS, 75 μm) and polydimethylsiloxane/divinylbenzene (PDMS/DVB, 65 μm), were used to evaluate the effect of fiber coating on the extraction efficiency of VOCs from wine vinegar samples. Figure S1 shows the typical total ion chromatograms (TIC) obtained using different fiber coatings with 5 mL of vinegar sample during 15 min at 40 °C under constant magnetic stirring (700 rpm).

**Figure 2:**
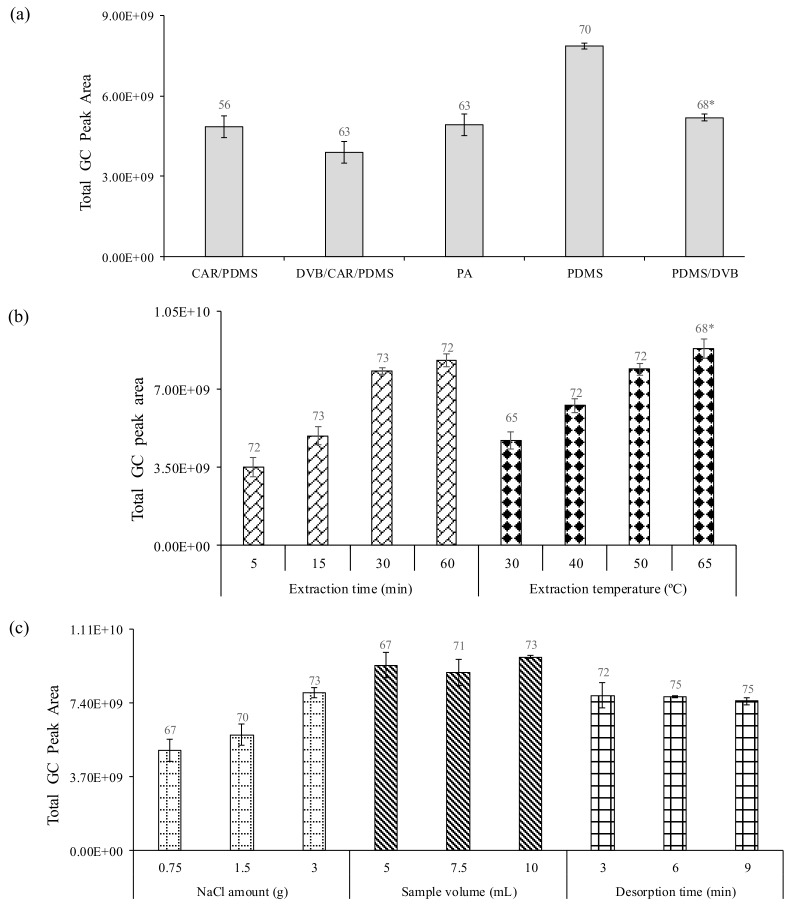
Effect of (**a**) fiber coating; (**b**) extraction time and extraction temperature; and (**c**) ionic strength, sample volume, desorption time on the volatile compounds extraction efficiency from wine vinegar. Error bars represent mean standard error (*n* = 3 for each data point). * Number of identified compounds.

A total of 70 VOCs were identified using a PDMS fiber, while with the PDMS/DVB, CAR/PDMS, DVB/CAR/PDMS and PA fibers, were detected 68, 56, 63 and 63, respectively (Figure 2a). Each extraction was performed in triplicate and the precision (% RSD) was lower than 15%. According to the total GC peak area and obtained reproducibility, the most suitable fiber for the extraction of VOCs was the PDMS followed by the PDMS/DVB, CAR/PDMS, PA and DVB/CAR/PDMS. Therefore, the PDMS fiber was selected for further assays.

#### 2.1.2. Extraction Time

Time affects the mass transfer of the analytes between the three system phases in HS-SPME technique, mainly determined by the agitation rate and the partition coefficient of the analyte between fiber coating and sample matrix [28]. SPME has a maximum sensitivity at the equilibrium state, however full equilibration is not necessary for accurate and precise analysis. The extraction time for the PDMS fiber was assayed by plotting the GC response vs. the extraction time to obtain the partition equilibrium profile (Figure 2b). The HS-SPME extraction efficiency increase with extraction time. After 30 min no remarkable differences were observed in terms of GC response as well as number of identified VOCs. The highest % RSD values, were observed at 5 and 15 min. These extraction times are too short to allow the system reach the equilibrium. Hence, an extraction time of 30 min was selected, since in terms of total GC peak areas, number of identified VOCs and precision, achieved the best performance.

#### 2.1.3. Extraction Temperature

Temperature has a significant effect on the extraction kinetics by HS-SPME. An increase in extraction temperature causes an increase in extraction rate and a simultaneously decrease in the distribution constant. Therefore, an adequate temperature which provides suitable sensitivity and extraction rate should be used [27,28]. The extraction temperature effect was investigated by sampling wine vinegar at different temperatures, 30, 40, 50 and 65 °C using the PDMS fiber, 5 mL of vinegar sample for 30 min under constant magnetic stirring (800 rpm). As can be seen in Figure 2b, the best extraction efficiency was obtained at 65 °C in terms of total GC peak area. In comparison with 50 °C, the obtained results showed higher % RSD whilst the number of VOCs identified was lower (68 VOCs). This decrease could be explained by VOCs degradation due to the higher temperature applied in the extraction procedure [29]. Taking into account the obtained results, 50 °C was selected as extraction temperature for further experiments.

#### 2.1.4. Ionic Strength

The suitability of the HS-SPME for the extraction of VOCs depends on the transfer of the analyte from sample to the gaseous phase and therefore to the fiber. This process can be optimized by the increase of the ionic strength [28]. Different NaCl amounts (0.75, 1.5 and 3 g) were evaluated using a PDMS fiber with 5 mL of wine vinegar for 30 min at 50 °C under constant magnetic stirring (800 rpm). As can be seen in Figure 2c, the total GC peak area as well as the number of identified VOCs increased as the NaCl amount increase, from 0.75 to 3g. Based on the obtained results, 3 g of NaCl was used to optimize the medium ionic strength and therefore promoting the “salting-out” effect, maximizing the extraction efficiency.

#### 2.1.5. Sample Volume

The amount of sorbed analyte by the SPME fiber coating can be influenced by sample volume. It was expected, that the transition temperature would be lower if the headspace volume was higher. With a constant vial size, the sample volume should be reduced to increase the headspace volume, without affecting the extraction efficiency of SPME [30,31]. In order to evaluate the effect of sample volume on HS-SPME extraction efficiency, a sample volume ranging, from 5 to 10 mL, was investigated using a PDMS fiber, 3 g of NaCl for 30 min at 50 °C under constant magnetic stirring (800 rpm). The sample volume of 10 mL was selected for the analysis since a total of 73 VOCs were identified, whilst by using 5 and 7.5 ml of sample volume, were identified 66 and 71 VOCs, respectively (Figure 2c). In addition, the % RSD for a volume of 5 and 7.5 mL of sample is high compared to 10 mL. In terms of total GC peak area no remarkable differences were observed. Thus, 10 mL of sample was selected for the further assays.

#### 2.1.6. Desorption Time

Efficient thermal desorption of analytes in a GC injection port depends on the analytes volatility, the thickness of the fiber coating, injection depth, injection temperature and extraction time. Desorption time was evaluated in the range of 3 to 9 min. As can be seen in Figure 2c, no remarkable differences in terms of GC peak area were observed for all tested desorption times using a PDMS fiber with 10 mL of vinegar sample and 3 g of NaCl for 30 min at 50 °C under constant magnetic stirring (800 rpm). Moreover, 75 VOCs were identified using 6 and 9 min of desorption time. Hence, 6 min was chosen for desorption of the VOCs from vinegar samples.

### 2.2. Characterization of Volatile Signature of WBAV by Maceration Using HS-SPME/GC-MS

Aroma is an important quality criteria for vinegars. Thus, the identification of VOCs responsible for its aroma is considered to be a key factor for quality and authentication control [4,19,22]. HS-SPME/GC-MS method was applied to establish the volatile signature of wine (control) and WBAV (banana, passion fruit, apple, pennyroyal). A total of 103 VOCs were identified in vinegar samples (Table 1) belonging to different chemical families, namely ethyl esters (37), alcohols (20), acids (10), terpenoids (23), carbonyl compounds (six), lactones (five) and volatile phenols (two). As far as we know, 34 of these VOCs are reported herein for the first time in vinegars (Table 1). These VOCs have been already identified by matching the obtained mass spectra with the reference compounds spectra in NIST Mass Spectral Search Program with a resemblance percentage above 80% and by comparison of the RIs calculated (KI_calc_) with the values reported in the literature (KI_lit_) for polyethylene glycol (or equivalent) column in vinegar samples using HS-SPME/GC-MS (Table 1). A range between 0 and 36 (|KI_calc_ − KI_lit_|) was obtained for KI_cal_ compared to the KI_lit_ reported in the literature for one dimensional GC with polyethylene glycol GC column or equivalent. This difference in KI is acceptable (<5%) taking into account that the literature data is obtained from a large range of GC stationary phases (several commercial GC columns are composed of polyethylene glycol or equivalent stationary phases).

**Figure 3:**
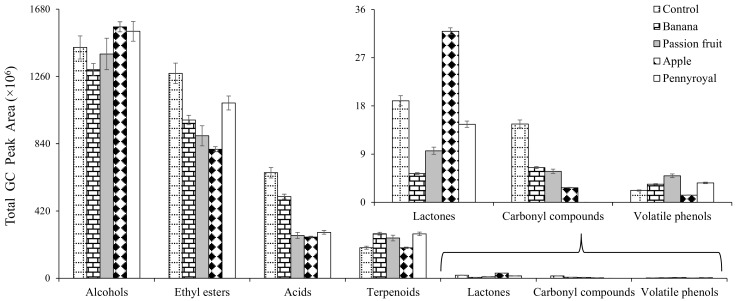
Total GC-MS peak area of chemical families identified in wine (control) and WBAV (banana, passion fruit, apple, pennyroyal) vinegars.

Forty-six VOCs were common in all vinegar samples analyzed, namely 17 ethyl esters, 11 alcohols, nine terpenoids, six acids, two lactones and one volatile phenol (Table 1). Moreover, some new VOCs, not found in wine vinegar (control)—mainly ethyl esters (e.g., ethyl 3-hexenoate) and terpenoids (e.g., limonene oxide, bornyl acetate, menthol)—appear in WBAV, as result of the maceration process. However, their contribution to the volatile signature was different for each vinegar sample, according to the raw material used. Table 1 shows the mean of GC peak area for each WBAV under study. The distribution of VOCs, according to its chemical family, is represented in Figure 3. Alcohols were the predominant chemical family in the studied vinegars. On average, the % RPA of alcohols was higher in WBAV, ranging from 42.10 (banana vinegar) to 54.91 % (apple vinegar), than in wine vinegar (control, 39.97%). Ethanol, 3-methylbutanol and 2-phenylethanol were markedly the most abundant alcohols identified in all vinegar samples. The presence of these VOCs in vinegar samples can contribute positively with flower, fruity and sweet notes for sensory properties. Due to its importance in terms of quality and acceptance by consumers and because there are no published reports on this subject, the sensory expression of WBAV is being evaluated in an ongoing work aiming to define its main odor descriptors. In addition the limiar of olfactive perception of the most significant volatiles will be established in order to evaluate its contribution to the sensory characteristics of vinegars. 2-Hexanol, 2-heptanol, 1-octanol, 1-octne-3-ol, 2-nonanol, 2,3-butanediol isomer and methionol were detected in all WBAVs, with the exception on pennyroyal vinegar. Moreover, 2-butanol was not detected in WBAV macerated with passion fruit and apple.

Ethyl esters are qualitatively the main chemical family (37 VOCs), and represent the second major contribution to the WBAV volatile signature (from 28.18 (apple vinegar) to 35.48% (wine vinegar)). This chemical family contribute positively to the general quality of WBAV being responsible for their fruity and floral sensory properties. Among the ethyl esters, Table 1, ethyl hexanoate, ethyl octanoate, ethyl decanoate, isoamyl acetate and ethyl acetate are the most abundant, accounting 86.01% and 80.82% of total volatile profile of wine vinegar (control) and WBAV, respectively. Heptyl acetate (0.04%) was only detected in wine-based vinegar (control), whereas, methyl octanoate (banana and passion fruit vinegars), ethyl 2-hydroxyisovalerate (banana and apple vinegars), isopentyl hexanoate (passion fruit and pennyroyal vinegars) and butyl octanoate (banana and apple vinegars) were detected only in WBAV. This fact suggest that these compounds come from raw material contributing to the flavor enrichment of macerated vinegars.

Fatty acids contributed with 18.26% and 10.95% for total volatile signature of wine vinegar and wine-based macerated vinegars, respectively. The lowest contribution was observed in pennyroyal (8.89%), followed by apple (8.99%), passion fruit (9.44%), banana (16.48%) and wine (18.26%) vinegar. Acetic, octanoic and decanoic acids were predominant in the studied vinegar samples. Its contribution to the total volatile profile was highest in wine vinegar (16.02%), followed by macerated wine vinegars, banana (14.69%), passion fruit (8.65%), apple (8.11%) and pennyroyal (7.60%).

Terpenoids represent an excellent example of aromatic compounds from varietal origin, which may be used for authentication and/or typicality of food matrices. The WBAV macerated with banana seems to be richest in terpenoids (8.99%), followed by passion fruit (8.88%), pennyroyal (8.62%), apple (6.65%) and control (5.29%). Moreover, linalool oxide isomer, menthone, limonene oxide, dihydrocarveol, bornyl acetate, linalyl acetate and menthol, were only detected in WBAVs (Table 1) suggesting the contribution of the maceration to the enrichment of wine vinegars. Limonene and α-terpeniol were the predominant terpenoids accounting 78.95%, 51.42%, 57.41%, 75.44% and 62.05% for the total terpenoids in wine vinegar (control), and macerated vinegars with banana, passion fruit, apple and pennyroyal, respectively. From a healthy point of view, several studies demonstrated the effectiveness of limonene and α-terpineol against some types of cancer including gastric, mammary, pulmonary adenoma and liver, as well as effective in relieving gastroesophageal reflux disorder and occasional heartburn [34,35].

Carbonyl compounds were not detected in vinegars macerated with pennyroyal (Table 1), whereas octanal was only detected in vinegars macerated with passion fruit. Undecanone and dodecanal were only detected in wine vinegar (control) and vinegars macerated with passion fruit. This chemical group contribute with 0.41% for total volatile profile of wine vinegar (control), whereas in macerated vinegars its contribution ranged from 0.10 (apple) to 0.21% (banana).

In wine vinegar, the lactones contribute with 0.53% for the total volatile profile, whereas in vinegars macerated with banana, passion fruit, apple and pennyroyal, its contribution was 0.17%, 0.34%, 1.12% and 0.45%, respectively. γ-Octalactone was only detected in wine vinegar (control), whereas γ-decalactone and δ-decenolactone were detected in all WBAV. From a sensorial point of view, lactones showed a positive contribution to aroma, with caramel, fruity, sweet and coconut-like notes [36].

### 2.3. Multivariate Analysis

The GC peak area of 103 VOCs (GC–MS data set) identified in wine vinegar (control) and WBAV were auto-scaled and submitted to PCA and HCA to search for the main sources of variability as well as to characterize and differentiate the vinegars based on their volatile signature. 

**Figure 4:**
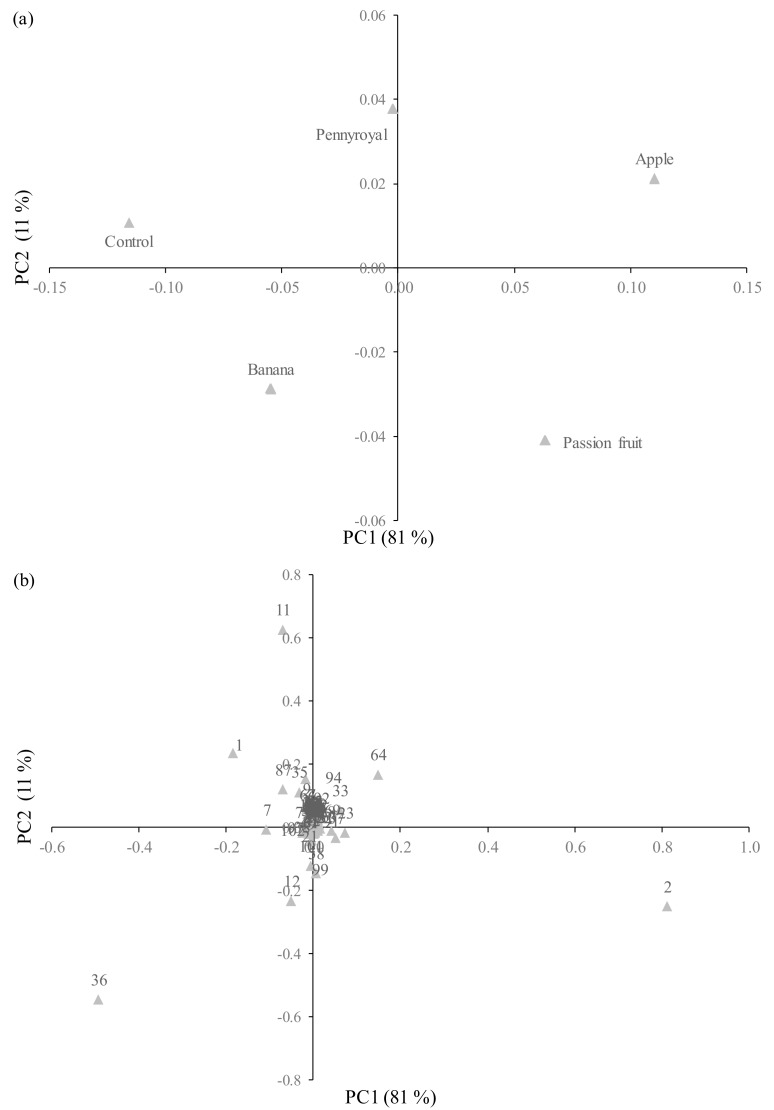
(**a**) Principal components (PC) PC1 × PC2 of scores scatter plot of wine (control) and WBAV vinegars; (**b**) Loading plot of the main source of variability between volatile profile and wine vinegars—derived products by maceration with fruits and plant (attribution of the peak number shown in Table 1).

PCA was used as the first exploratory step in our research to better understand the usefulness of the volatile signature to define and differentiate wine vinegar—derived products by maceration with fruits and medicinal herb. The PCA score scatter plot of the two first principal components, which explain 92% of total variability of GC-MS data, is represented in Figure 4a. The loadings of each VOCs (variables) that explain the differentiation among vinegar samples are shown in Figure 4b. According to results obtained by PCA, apple vinegar (PC1 and PC2 positive) is mainly characterized by the presence of ethyl decanoate (64), whereas banana vinegar (PC1 and PC2 negative) is essentially characterized by acetic acid (36). Wine vinegar (control) and pennyroyal vinegars were projected in PC1 negative and PC2 positive being essentially characterized by ethyl acetate (1) and 3-methylbutanol (11), respectively. The passion fruit vinegar (PC1 positive and PC2 negative) is described by ethanol (2).

**Figure 5:**
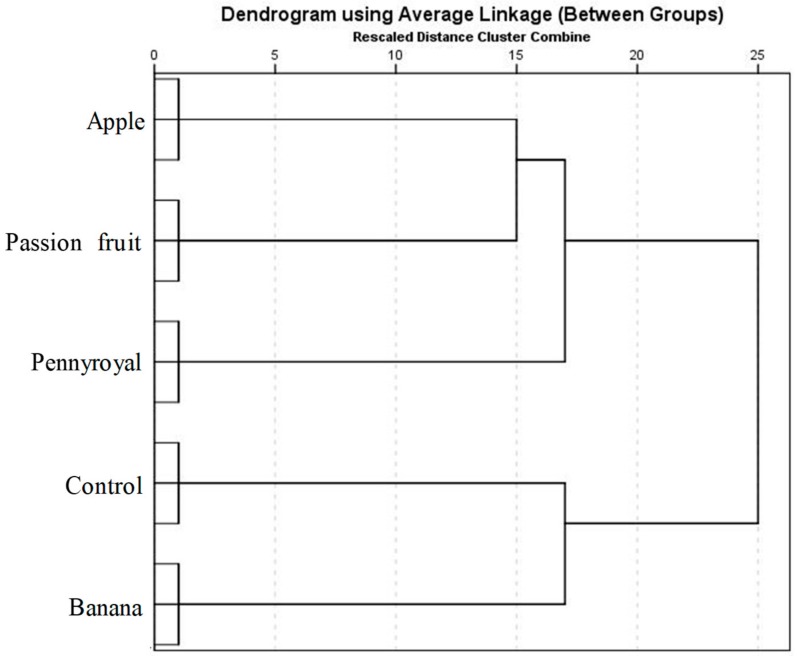
Dendrogram for the HCA results using Ward’s cluster algorithm of the volatile profile obtained from wine (control) and WBAV vinegars. The Square Euclidean distances are shown on the *x*-axis.

The results of HCA for vinegar samples based on the GC–qMS data set is shown in Figure 5. The horizontal lines in the obtained dendrogram represent the similarity values between pairs of samples, between a sample and a group of samples and between sample groups, and the vertical lines the studied samples. The vinegar samples were firstly grouped into two clusters, i.e., cluster A (wine vinegar and banana vinegars) and cluster B (passion fruit, apple and pennyroyal vinegars). Moreover, cluster A could also be further grouped into sub-clusters, such as A1 (wine vinegar) and A2 (banana vinegar). These results are in agreement with those obtained by PCA, and it is possible to find that these clusters were evidently related to vinegar sample as well as to volatile signature.

## 3. Materials and Methods

### 3.1. Chemicals and Materials

All chemicals used in this study were of analytical quality. Sodium chloride (NaCl, 99.5%) used to adjust the ionic strength, was purchased from Panreac (Barcelona, Spain). VOCs standards used for identification of target compounds with purity >98% were purchased from Sigma-Aldrich (Madrid, Spain), Acros Organics (Geel, Belgium) and Fluka (Buchs, Switzerland). The individual stock solutions were prepared in ethanol (concentration of 1000 mg/L) and stored at 4 °C. Helium (Air Liquide, Miraflores, Algés, Portugal) was used as GC carrier gas. The glass vials, SPME fibers, and SPME holder for manual sampling were purchased from Supelco (Bellefonte, PA, USA). The Kovats index (KI) was calculated through injection of a series of C_8_ to C_20_ straight-chain *n*-alkanes (concentration of 40 mg/L in *n*-hexane) was supplied by Fluka.

### 3.2. Vinegar Samples

The WBAVs were obtained according to Procedure I in Figure 1. For this purpose, individual maceration of wine vinegar with different fruits and the medicinal herb was carried out. The vinegar used to perform the maceration was supplied by a local winery. The fruits used for maceration, banana (*Musa sapientum* L.), passion fruit (*Passiflora edulis* L.), apple (*Malus domestica* Borkh.) and the medicinal herb (pennyroyal (*Mentha pulegium* L.)), were purchased from a local market at optimal maturity and health stage. Samples were washed and dried to remove possible contaminants. 

To promote the enrichment of volatile profile through maceration process, dark glass containers (1000 mL) were filled with 750 mL of wine vinegar, to which small pieces (50 g) of banana, passion fruit, apple and pennyroyal were added. All dark glass containers were continuously stirred during 7 days at room temperature (25 ± 1 °C). The wine vinegar without maceration was used as control vinegar. Each assay was performed in triplicate.

### 3.3. HS-SPME Procedure

To improve the extraction efficiency of HS-SPME several parameters such as: fiber coating, extraction time (5, 15, 30, 60 min), extraction temperature (30, 40, 50, 60 °C), sample volume (5, 7.5, 10 mL), ionic strength (0.75, 1.5, 3 g) and desorption time (3, 6, 9 min) were tested and optimized using an univariate experimental design.

For fiber screening, five commercial fibers, namely carboxen/polydimethylsiloxane (CAR/PDMS, 75 µm), divinylbenzene/carboxen/polydimethylsiloxane (DVB/CAR/PDMS, 50/30 µm), polyacrylate (PA, 85 µm), polydimethysiloxane (PDMS, 100 µm) and polydimethylsiloxane/divinylbenzene (PDMS/DVB, 65 µm) were evaluated. Before daily analyses each fiber was conditioned for 15 min at 250 °C.

For HS-SPME optimization, a wine-based vinegar (Control) was used. For each extraction, 10 mL of sample was transferred to a 20 mL amber glass vial with 3 g of NaCl. A magnetic stirrer bar was added. The vial, tightly capped with a PTFE-faced silicone septum, was placed in a thermostatic block with a constant magnetic stirring (800 rpm). The SPME fiber was exposed into the headspace during 30 min at 50 °C. Subsequently, after extraction the fiber was withdrawn into the holder needle, removed from the vial and immediately introduced into the GC injector port for 6 min at 250 °C for thermal desorption of the analytes. All assays were performed in triplicate.

### 3.4. GC-MS Conditions

An Agilent 6890N gas chromatograph system (Palo Alto, CA, USA) combined with an Agilent 5975 quadrupole inert mass selective detector and a splitless injector was used. Chromatographic separations were performed using a BP-20 (30 m × 0.25 mm i.d. × 0.25 µm film thickness) fused silica capillary column supplied by SGE (Darmstadt, Germany) with helium (Helium N60, Air Liquide) as carrier gas at a flow rate of 1 mL/min (column-head pressure: 13 psi). An insert of 0.75 mm i.d. was used and the injector temperature was fixed to 250 °C. The temperature program was programmed as follows: initial temperature 40 °C and a ramp of 3 °C min^−1^ to 220 °C and maintaining a constant temperature for 10 min until the end. The manifold, GC-MS interface and quadrupole temperatures were held at 180, 220 and 180 °C, respectively. MS detection was performed in full scan, the ion energy used for the electron impact (EI) was 70 eV and the source temperature was 180 °C. The electron multiplier was set to the auto tune procedure. The mass acquisition range, made in full scan mode, was 30–300 m/z, 1.9 spectra s^−1^. VOCs identification was achieved by the following ways:

(i) comparison the GC retention times and mass spectra with those of the standard, when available;
(ii) all mass spectra were also compared with the data system library (NIST Mass Spectral Search Program v.2.0d software; NIST: Washington, DC, USA, 2005);
(iii) Kovats index (KI) values were determined according to the van den Dool and Kratz equation [37].

For the determination of the KI, a C_8_–C_20_ *n*-alkanes series was used, and the values were compared, when available, with values reported in the literature for similar columns [5,27,32,33].

### 3.5. Statistical Analysis

Chemometric tools, namely PCA and HCA, were applied to the auto-scaled areas of the identified VOCs in wine-based aromatic vinegars (WBAV), using SPSS version 23 Statistical Package for Windows (SPSS Inc., Chicago, IL, USA). Auto-scaling is a data pre-treatment process that makes variables of different scales comparable. Each variable is autoscaled separately by subtracting its mean value and dividing by its standard deviation. The HCA was processed employing Ward’s minimum variance algorithm method, and the distance was determined using squared Euclidean distances. The Ward’s algorithm consists in aggregating two clusters such that the growth of within-inertia is minimum, i.e., minimizing the reduction of the between-inertia, at each step of the algorithm. The within inertia characterizes the homogeneous of a cluster. The goal was to extract the main sources of variability and hence to help on the characterization of the dataset [38].

## 4. Conclusions

The optimized method based on the HS-SPME/GC-MS was revealed as a suitable tool to establish the volatile signature of WBAVs obtained by maceration with fruits and a medicinal herb. A total of 103 VOCs, belonging to different chemical families, were identified. In terms of % RPA values and VOCs identified differences were observed between wine vinegar and WBAV, as well as among WBAV. In the case of WBAV, the different contribution for total volatile profile could be explained by the fruit volatile signature used to macerate the wine vinegar (control). The fruit volatile signature can be affected by climatic conditions, sun exposition and agronomic practices. Higher alcohols, ethyl esters and fatty acids were the predominant chemical families found in all investigated vinegar samples. On the other hand, in qualitative terms, carbonyl compounds were not detected in pennyroyal vinegar, whereas ethyl 3-ethoxypropanoate, ethyl 3-hexenoate, methyl octanoate, ethyl 2-hydroxyisovalerate, isopentyl hexanoate, butyl octanoate, linalool oxide isomer, menthone, limonene oxide, dihydrocarveol, bornyl acetate, linalyl acetate, menthol and octanal were only detected in some WBAVs. From a sensorial point of view, the ethyl esters and terpenoids only detected in some WBAV showed a positive contribution to aroma with fruity, sweet, green, fresh, anise and berry notes to WBAV aroma, since they have low odor threshold (in order of µg/L). However, a future work will be done related to sensorial analysis of WBAV using HS-SPME/GC-O in order to define its odor descriptors and provide their distinct patterns based on their volatile signature depending on the raw material used in the maceration process. As far as we known, no studies are available related to sensorial analysis of WBAV and therefore it will be subject of a future publication.

The HS-SPME/GC-MS data combined with PCA and HCA provided a powerful tool to differentiate vinegar from WBAV based on volatile signature. This knowledge represents a suitable tool to guarantee the authenticity and genuineness of wine as well wine-based aromatic vinegars, to promote the production of vinegars with enhanced levels of volatile compounds. As far as we know, 34 VOCs were identified for the first time in vinegars. 

## Figures and Tables

**Table 1:** Volatile organic compounds (VOCs) identified in wine-based aromatic vinegars (WBAVs) by headspace solid-phase microextraction (HS-SPME)_PDMS_/GC-MS.

Peak Number	RT ^1^ (min)	KI_cal_^2^	KI_lit_^3^	Chemical Families	GC Peak Area × 10^6^ (RSD)									
					Wine Vinegar Control		Macerated Vinegars							
							Banana		Passion Fruit		Apple		Pennyroyal	
				*Ethyl esters*										
1	2.91	925	907	Ethyl acetate	300.22	(8)	197.65	(9)	122.23	(13)	113.89	(9)	247.35	(3)
4	4.68	1055	1028	Ethyl butanoate	16.03	(6)	- ^5^		9.93	(14)	3.29	(4)	-	
5	5.29	1081	1050	Ethyl 3-methylbutanoate	15.18	(6)	16.36	(17)	3.31	(13)	8.12	(4)	-	
7	6.51	1125	1120	Isoamyl acetate	163.77	(15)	169.1	(9)	71.31	(3)	80.64	(2)	124.8	(16)
12	10.04	1222	1220	Ethyl hexanoate	227.53	(9)	113.51	(8)	195.43	(12)	84.73	(20)	128.32	(14)
16	12.41	1285	1304	Ethyl 3-ethoxypropanoate ^4^	-		9.98	(8)	-		0.58	(11)	2.16	(18)
17	12.67	1291	1292	Ethyl 3-hexenoate^4^	-		6.44	(12)	1.06	(7)	0.87	(9)	4.62	(6)
18	13.23	1305	1300	3-Hexen-1-ol acetate isomer	3.14	(4)	0.94	(15)	-		2.17	(11)	-	
21	13.87	1320	1305	Ethyl 2-hexenoate	14.86	(6)	0.87	(9)	1.27	(19)	1.10	(4)	2.81	(5)
22	14.67	1339	1358	Ethyl lactate	21.95	(14)	42.24	(16)	36.08	(4)	28.80	(14)	51.13	(1)
25	15.53	1343	1350	Hexyl acetate	22.48	(17)	29.76	(11)	18.26	(9)	16.00	(2)	12.48	(13)
26	15.58	1359	-	Heptyl acetate ^4^	0.52	(15)	-		-		-		-	
29	16.13	1370	-	2-Ethylhexyl acetate ^4^	2.99	(9)	2.40	(3)	-		-		-	
30	16.16	1372	1389	Methyl octanoate	-		1.84	(7)	0.54	(8)	-		-	
34	17.82	1405	1394	Ethyl 2-hydroxyisovalerate	-		2.46	(11)	-		0.13	(13)	-	
35	18.16	1416	1414	Ethyl octanoate	296.15	(4)	182.15	(3)	181.16	(10)	194.75	(2)	208.86	(11)
37	19.19	1441	-	Isopentyl hexanoate	-		-		77.49	(8)	-		82.04	(4)
47	22.33	1518	1483	Ethyl 3-hydroxybutanoate	2.65	(13)	-		3.09	(1)	1.32	(4)	-	
49	22.61	1526	1551	2-Ethyl hydroxycaproate ^4^	6.45	(19)	8.08	(6)	2.62	(14)	0.42	(11)	6.02	(8)
51	23.04	1536	1533	Hexyl butanoate ^4^	1.21	(7)	-		-		1.74	(3)	-	
64	26.58	1617	1636	Ethyl decanoate	112.62	(3)	132.17	(14)	110.55	(5)	211.67	(4)	138.31	(2)
65	26.85	1625	1610	Butyl octanoate ^4^	-		0.27	(20)	-		1.47	(16)	-	
66	27.35	1639	1648	Ethyl benzoate	6.63	(15)	6.26	(8)	5.83	(11)	6.65	(1)	9.15	(4)
68	28.07	1659	1680	Diethyl succinate	25.99	(5)	23.95	(15)	15.49	(4)	18.84	(2)	23.72	(5)
71	29.34	1693	1664	Ethyl 3-hydroxyhexanoate	1.27	(4)	1.02	(9)	0.20	(12)	0.21	(20)	0.98	(5)
75	31.42	1754	1755	Phenylmethyl acetate	2.78	(6)	-		-		0.27	(12)	-	
76	32.07	1773	1775	Ethyl benzeneacetate	11.12	(14)	7.44	(8)	4.61	(8)	3.95	(7)	11.51	(7)
77	32.47	1784	-	Dibuthyl succinate ^4^	0.31	(3)	-		-		1.41	(2)	-	
78	32.73	1791	1798	Methyl 2-hydroxybenzoate	0.63	(17)	6.07	(10)	-		-		4.23	(2)
80	34.32	1838	1837	Ethyl dodecanoate	10.17	(11)	12.15	(16)	13.26	(12)	8.75	(3)	12.55	(15)
82	34.99	1857	1821	Benzyl propanoate ^4^	7.54	(4)	1.32	(14)	9.26	(8)	11.71	(15)	5.61	(2)
83	35.58	1873	1849	Benzyl butanoate ^4^	0.48	(13)	-		1.37	(11)	0.58	(16)	-	
84	35.87	1880	1883	2-Phenylethyl acetate	1.00	(11)	5.73	(5)	1.19	(8)	-		6.34	(13)
85	36.47	1881	1880	Citronellyl valerate ^4^	0.36	(1)	1.20	(21)	1.35	(10)	0.33	(6)	8.87	(3)
90	39.66	1970	1974	Methyl jasmonate ^4^	0.98	(2)	0.81	(4)	-		0.26	(12)	-	
95	43.13	2193	2189	Phenylethyl benzoate ^4^	1.82	(3)	3.52	(13)	1.81	(18)	0.82	(4)	2.20	(11)
100	48.08	2308	2301	Methyl hexadecanoate	0.41	(6)	1.99	(12)	0.45	(6)	-		0.53	(14)
				*Alcohols*										
2	3.15	968	972	Ethanol	773.10	(10)	845.14	(11)	1040.94	(16)	1099.28	(12)	946.25	(6)
3	4.66	1074	1099	2-Butanol	12.75	(6)	11.75	(19)	-		-		14.45	(2)
6	6.12	1113	1112	2-Methyl-1-propanol	23.13	(12)	13.42	(9)	9.14	(12)	14.55	(17)	19.72	(8)
8	7.87	1165	1176	2-Hexanol	2.75	(7)	3.25	(10)	1.86	(12)	2.19	(15)	-	
11	9.51	1206	1206	3-Methylbutanol	436.65	(8)	262.37	(13)	209.63	(9)	301.03	(7)	399.42	(3)
20	13.51	1312	1332	2-Heptanol ^4^	0.23	(4)	1.23	(6)	1.01	(3)	1.61	(8)	-	
23	15.15	1350	1360	1-Hexanol	23.03	(10)	33.89	(13)	69.49	(3)	55.64	(10)	63.41	(3)
24	15.23	1352	1386	3-Hexenol isomer	0.22	(18)	0.47	(9)	0.26	(2)	0.77	(19)	0.41	(15)
27	15.81	1364	1379	3-Ethoxypropanol ^4^	0.14	(10)	1.82	(6)	1.29	(14)	-		-	
28	16.11	1371	1391	3-Hexenol isomer	5.78	(3)	1.83	(16)	2.01	(11)	8.56	(13)	6.49	(4)
33	17.69	1383	1388	1-Octanol	10.25	(4)	6.01	(16)	10.23	(1)	5.63	(2)	-	
42	20.48	1475	1465	1-Octen-3-ol	7.12	(1)	7.91	(2)	9.67	(9)	0.32	(3)	-	
46	21.71	1503	1487	2-Ethylhexanol	2.56	(5)	2.54	(13)	-		8.17	(9)	-	
48	22.43	1521	1546	2,3-Butanediol isomer	5.06	(3)	14.92	(12)	1.79	(4)	0.29	(13)	10.03	(1)
52	23.23	1540	1535	2-Nonanol ^4^	0.53	(8)	1.91	(3)	4.37	(7)	3.13	(8)	-	
56	23.93	1556	1583	2,3-Butanediol isomer	9.44	(11)	5.73	(15)	1.52	(8)	0.27	(11)	-	
72	29.65	1701	1723	Methionol	1.21	(2)	1.39	(19)	1.35	(5)	1.66	(8)	-	
73	31.28	1750	1765	1-Decanol ^4^	1.60	(12)	5.82	(12)	1.08	(17)	2.22	(17)	8.32	(9)
87	37.94	1898	1925	2-Phenylethanol	124.77	(5)	78.46	(12)	27.58	(7)	63.08	(6)	68.75	(6)
89	39.24	1956	1952	Tridecanol ^4^	0.67	(7)	2.14	(12)	6.68	(10)	0.99	(12)	4.35	(2)
				*Terpenoids*										
9	8.38	1178	1182	Limonene	101.05	(1)	82.77	(6)	79.66	(12)	78.23	(3)	104.46	(8)
10	8.76	1187	1214	Eucalyptol	1.11	(4)	4.21	(21)	2.13	(2)	-		2.15	(4)
13	11.18	1254	1233	Sabinene	2.22	(15)	6.03	(2)	1.19	(15)	-		-	
19	13.38	1308	1337	Rose oxide isomer ^4^	8.25	(3)	8.31	(2)	9.78	(12)	9.23	(1)	9.77	(4)
32	16.74	1392	1421	Linalool oxide isomer	-		-		1.45	(12)	0.33	(10)	8.76	(6)
38	19.22	1443	1449	Dihydrolinalool ^4^	1.32	(8)	9.83	(14)	1.37	(20)	2.61	(12)	-	
39	19.4	1448	1467	Linalool oxide isomer	-		3.32	(14)	0.96	(7)	0.18	(6)	-	
40	19.97	1462	1474	Menthone ^4^	-		8.42	(9)	4.19	(2)	-		14.19	(14)
41	20.16	1467	1467	Limonene oxide	-		12.48	(15)	6.40	(7)	9.81	(3)	7.15	(3)
44	21.47	1498	1531	Vitispirane I	5.61	(8)	6.21	(15)	3.95	(1)	4.95	(5)	6.57	(5)
45	21.57	1501	1534	Vitispirane II	12.14	(18)	4.25	(16)	4.31	(2)	3.13	(11)	3.36	(9)
50	22.88	1530	1537	Linalool	1.02	(8)	3.35	(7)	6.15	(4)	2.15	(4)	1.98	(4)
53	23.28	1541	1538	Dihydrocarveol ^4^	-		11.61	(3)	14.14	(5)	-		21.68	(14)
55	23.72	1551	1580	Bornyl acetate ^4^	-		4.89	(4)	1.38	(5)	2.81	(8)	1.18	(15)
58	24.44	1559	1569	Linalyl acetate	-		26.39	(7)	28.71	(11)	-		-	
59	24.83	1575	1574	Fenchyl alcohol ^4^	0.58	(2)	0.73	(8)	0.42	(9)	0.78	(4)	1.91	(20)
62	26.27	1610	1626	Menthol ^4^	-		5.94	(16)	5.66	(7)	5.40	(2)	7.62	(6)
63	26.34	1618	1632	Pulegone ^4^	2.84	(13)	-		2.75	(12)	-		-	
69	28.58	1673	1669	α-Terpeniol	49.62	(12)	60.22	(9)	64.55	(9)	65.23	(2)	67.71	(3)
74	31.38	1753	1762	Citronellol	0.94	(13)	2.73	(4)	-		-		1.72	(12)
81	34.59	1844	1840	Geranylacetone	4.16	(15)	15.36	(19)	10.45	(5)	4.24	(8)	12.76	(13)
91	39.86	1981	2009	Nerolidol	0.76	(8)	0.82	(3)	1.21	(4)	-		2.43	(4)
93	41.16	2125	2134	α-Cadinol	4.23	(9)	0.20	(13)	0.37	(2)	1.09	(7)	2.06	(15)
				*Carbonyl compounds*										
14	12.05	1276	1276	Octanal	-		-		0.52	(7)	-		-	
25	12.18	1278	1272	3-Hydroxybutan-2-one	3.01	(8)	2.71	(5)	0.75	(2)	-		-	
31	16.29	1374	1378	Nonanal	1.22	(7)	1.58	(7)	1.99	(10)	1.27	(7)	-	
43	20.81	1483	1484	Decanal	1.69	(8)	2.19	(13)	1.90	(2)	1.47	(8)	-	
57	24.08	1571	1543	Undecanone ^4^	2.72	(12)	-		0.21	(3)	-		-	
70	29.21	1689	1700	Dodecanal ^4^	5.99	(3)	-		0.94	(6)	-		-	
				*Acids*										
36	18.51	1425	1407	Acetic acid	422.11	(18)	347.48	(12)	139.85	(9)	136.06	(11)	122.36	(14)
54	23.52	1547	1572	2-Methylpropanoic acid	1.52	(14)	2.66	(7)	-		-		-	
61	25.97	1600	1619	Butanoic acid	3.77	(1)	8.09	(15)	0.92	(4)	0.44	(9)	1.25	(7)
67	27.56	1645	1665	Isovaleric acid	19.86	(13)	9.15	(12)	2.61	(7)	6.63	(11)	16.27	(5)
79	34.29	1837	1850	Hexanoic acid	37.85	(11)	32.83	(4)	17.25	(8)	15.06	(5)	21.49	(10)
86	36.69	1937	1962	2-Hexenoic acid isomer ^4^	0.23	(4)	0.68	(16)	0.92	(13)	-		1.68	(7)
94	41.82	2098	2083	Octanoic acid	83.66	(7)	41.12	(13)	34.35	(3)	53.22	(2)	76.83	(12)
101	48.56	2321	2317	Decanoic acid	71.71	(6)	65.85	(15)	70.36	(5)	42.58	(2)	45.52	(19)
102	54.81	2340	2310	Hydrocinnamic acid	10.63	(10)	1.02	(16)	0.81	(7)	2.43	(5)	-	
103	67.81	2392	2407	Undecylic acid ^4^	7.18	(8)	0.91	(15)	-		0.56	(8)	0.62	(9)
				*Lactones*										
60	25.71	1594	1618	Butyrolactone	0.94	(5)	-		-		0.19	(19)	-	
88	37.95	1936	1912	γ–Octalactone ^4^	0.53	(14)	-		-		-		-	
92	40.63	2107	2103	γ–Decalactone ^4^	2.28	(17)	0.95	(8)	2.28	(2)	14.59	(2)	4.23	(4)
96	43.92	2218	2209	Wine lactone ^4^	3.99	(2)	-		-		1.27	(7)	2.60	(12)
99	45.44	2267	2241	γ-Decenolactone ^4^	11.22	(6)	4.43	(8)	7.34	(13)	15.90	(2)	7.75	(1)
				*Volatile phenols*										
97	44.22	2228	2205	4-Ethylphenol	1.82	(4)	1.71	(14)	1.22	(3)	-		0.79	(3)
98	45.13	2257	2250	Eugenol	0.39	(8)	1.68	(10)	3.73	(10)	1.35	(4)	2.83	(4)

^1^ Retention time (min); ^2^ Kovats index relative *n*-alkanes (C8 to C20) on a BP-20 capillary column; ^3^ Kovats index relative reported in literature for equivalent capillary column [5,27,32,33]; ^4^ Reported for the first time in vinegars; ^5^ Not detected.

## Supplementary Materials

Supplementary materials are available online.
