# Supplementary material for: Establishment of the Volatile Signature of Wine-Based Aromatic Vinegars Subjected to Maceration
Source: Molecules. 2018 Feb 23;23(2):499. doi: 10.3390/molecules23020499 (PMC6017499; doi:10.3390/molecules23020499)
Supplement: Supplementary file 1 [file molecules-23-00499-s001.pdf]

molecules

MDPI

SUPPLEMENTARY MATERIAL:

Establishment of the global volatile signature of wine-based aromatic vinegars subjected to maceration

Rosa Perestrelo [1], Catarina L. Silva [1], Pedro Silva [1], José S. Câmara [1,2,*]

[1] CQM – Centro de Química da Madeira, Universidade da Madeira, Campus da Penteada, 9020-105 Funchal, Portugal (rmp@uma.pt; cgsluis@uma.pt; pedro_dasilva@hotmail.com)

[2] Departamento de Química, Faculdade de Ciências Exatas e Engenharia, Universidade da Madeira, Campus da Penteada, 9020-105 Funchal, Portugal (jsc@uma.pt)

* Correspondence: jsc@uma.pt; Tel.: +351 291705112

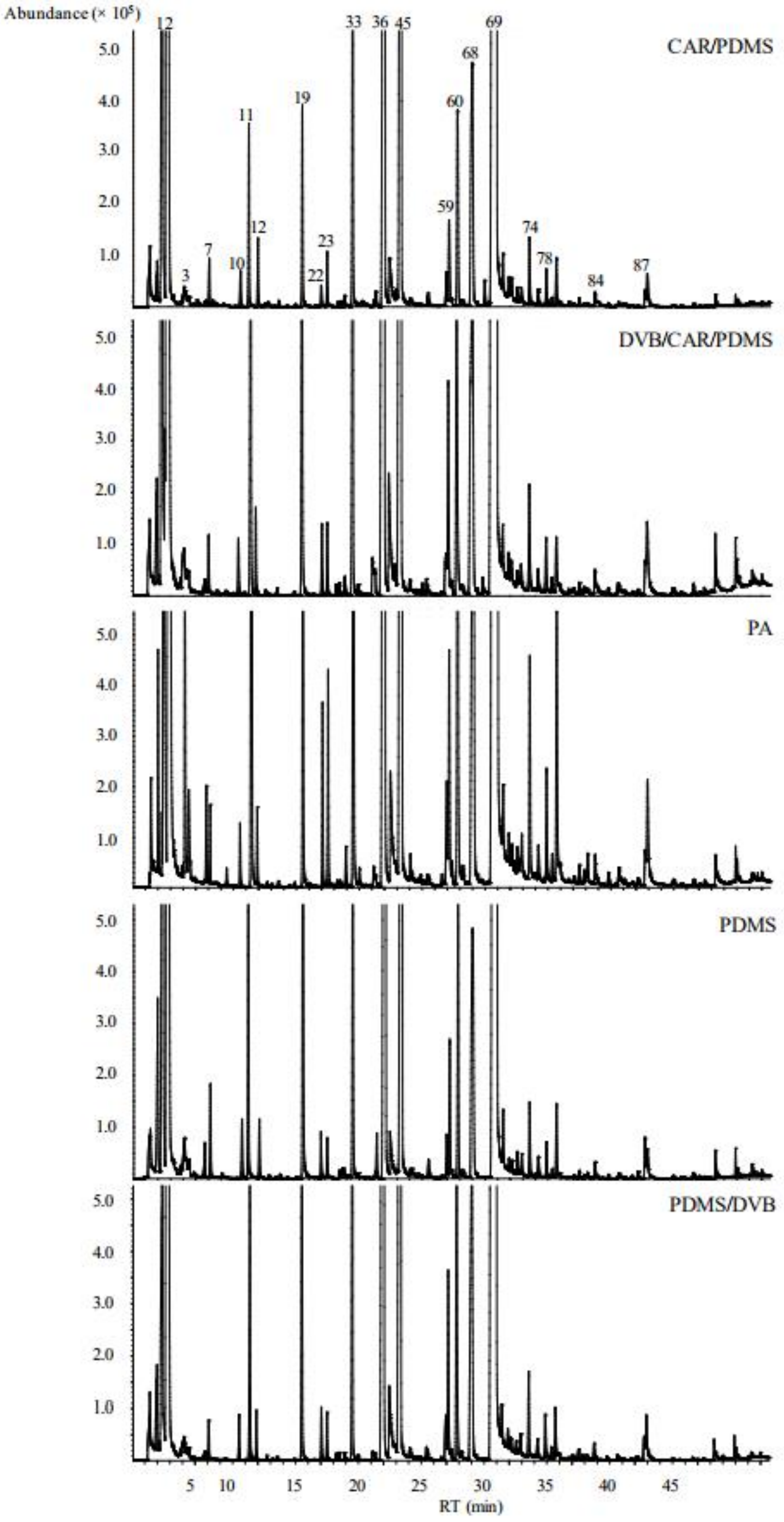

Figure S1. Total ion chromatograms obtained by HS-SPME/GC-MS analysis of wine vinegar (control) using different SPME fibers for 15 min at 40 °C under constant magnetic stirring (700 rpm). Attribution of the peak number shown in Table 1.
